# Parasites of veterinary importance from domestic animals in uMkhanyakude district of KwaZulu-Natal province

**DOI:** 10.4102/jsava.v91i0.2023

**Published:** 2020-07-13

**Authors:** Lehlohonolo S. Mofokeng, Oriel M. Taioe, Nico J. Smit, Oriel M.M. Thekisoe

**Affiliations:** 1 Unit for Environmental Sciences and Management, North-West University, Potchefstroom, South Africa; 2 National Zoological Garden, South African National Biodiversity Institute, Pretoria, South Africa

**Keywords:** *Babesia* sp., *Ehrlichia canis*, *Theileria* sp., *Trypanosoma* sp., *Toxoplasma gondii*.

## Abstract

This study investigated the occurrence and phylogenetic relationship of protozoan parasites and *Ehrlichia* infecting domestic animals from three municipalities in uMkhanyakude district of KwaZulu-Natal province, South Africa. A total of 208 blood samples collected from clinically healthy cattle, sheep, goats and dogs from uMkhanyakude district were examined by polymerase chain reaction (PCR) assays, using either genus or species-specific primers to determine the occurrence and phylogenetic relationship of various protozoan parasites and *Ehrlichia* of veterinary importance. A total of 5/109 (4.6%) cattle were PCR-positive for the presence of *Toxoplasma gondii*, 33/109 (30.3%) for *Babesia bovis,* 24/109 (22.02%) for *Babesia bigemina* and 20/109 (18.3%) for *Trypanosoma* sp., while 3/10 (30%) of sheep were PCR-positive for *Theileria ovis* and none of the goats were positive for any of the detected pathogens. The co-infection of 4/109 (3.7%) *B. bovis* and *B. bigemina* was detected in cattle. Only *Ehrlichia canis* was detected in dogs with infection rate of 20/48 (41.7%). Sequences of PCR-positive isolates (*B. bovis, B. bigemina, E. canis, T. ovis* and *T. gondii*) showed that they were closely related to their relevant species from various countries. These findings have expanded our knowledge about the prevalence and phylogenetic similarity between protozoan parasites and *Ehrlichia* isolates of South African origin. To date, this is the first study in South Africa to detect *T. gondii* infections from cattle blood using PCR.

## Introduction

Protozoan and ehrlichial diseases are significant constraints to the production of livestock in sub-Saharan Africa. In South Africa, around 18% of livestock mortalities are because of protozoan diseases (Mtshali & Mtshali 2013). These diseases have substantial impact on the country’s economic security and poor communities who are dependent on livestock production, as they lead to losses of meat, wool, milk and manure (Perry & Sones 2007; Ringo et al. 2018). In sub-Saharan Africa, little information is available on their presence and distribution. Normally, protozoan parasite infection is thought to result from a complex interaction between pathogens, vectors, vertebrate host and the environment (Weny et al. 2017). Piroplasmosis, trypanosomosis, ehrlichiosis, hepatozoonosis and toxoplasmosis are among parasitic diseases that cause significant threat to the health of domestic animals. Various piroplasm species such as *Babesia bovis, Babesia bigemina, Babesia ovis, Babesia motasi, Babesia rossi, Babesia vogelli, Theileria ovis, Theileria lestoquardi, Theileria seperata* and *Theileria parva* have been described in small ruminants. These species are known to be causative agents of babesiosis and theileriosis, respectively (Ijaz et al. 2013; Mohammadi et al. 2017). In southern Africa, *B. bovis* and *B. bigemina* are two economically important species infecting cattle and have high prevalence in tropical and subtropical regions (Mtshali & Mtshali 2013), while *B. ovis* is known to be highly pathogenic in sheep with a mortality ranges of 30% – 50% (Ijaz et al. 2013; Ringo et al. 2018). Two species of canine *Babesia, B. rossi* and *B. vogelli*, are known to be endemic to South Africa (Matjila et al. 2004). The clinical signs of *B. vogelli* have not yet been estimated and this led to *B. rossi* being considered as the most prevalent species in South Africa as it causes severe, often fatal disease (Jacobson 2006).

The most pathogenic member *Theileria*, particularly in sheep, is known to be *T. lestoquardi*, while *T. ovis* is reported to be less pathogenic and usually causes subclinical infection albeit animals subjected to stress may develop significant illness (Durrani et al. 2011). On the contrary, *T. seperata* is regarded as non-pathogenic but can be fatal to immunocompromised animals or those that are newly introduced to endemic areas (Luo & Yin 1997; Ringo et al. 2018). Following the eradication of East coast fever, Corridor disease emerged as the most significant form of theileriosis in South African cattle. In areas where common grazing among cattle and infected buffalo occur and where there is an abundance of tick vector species (*Rhipicephalus appendiculatus* and *Rhipicephalus zambeziensis*), the disease still poses a serious threat (Uilenberg 1999).

Among the causal agents of chronic, debilitating, emaciating and usually fatal disease in domestic animals, *Trypanosoma* infections are major causative agents of alopecia, emaciation, lymphadenopathy and anaemia in domesticated animals (World Organization Of animal Health [OIE] 2013). However, the outcome of the infection varies among trypanosome species, livestock species and the virulence of the strains (Connor & Van den Bossche 2004). *Trypanosoma vivax, Trypanosoma simiae, Trypanosoma uniforme, Trypanosoma brucei brucei* and *Trypanosoma congolense* are important causative agents of animal African trypanosomosis, also known as nagana in Africa, with tsetse flies acting as biological vectors for the cyclic transmission of the disease in domesticated animals (Steverding 2008). This is attributed to their pathogenicity and effects on productivity (Trail et al. 1994; Wellde et al. 1989).

*Toxoplasma gondii* is a widespread global zoonotic protozoan parasite that infects a wide range of warm-blooded animals (Howe & Sibley 1995). Humans and animals acquire infection through ingestion of raw and undercooked infected meat that contains viable *Toxoplasma* tissue cyst or food and drink contaminated with *Toxoplasma* oocysts excreted from the faeces of infected felids. This makes toxoplasmosis the most important foodborne and waterborne parasitic disease (Bowie et al. 1997; Torgerson et al. 2015). Most animals infected with toxoplasmosis show no clinical manifestation of the disease, but the disease is known to be the leading cause of abortion in sheep.

*Ehrlichia canis* and *Hepatozoon canis* are causative agents of canine monocytic ehrlichiosis and canine hepatozoonosis, respectively. The main vector of both pathogens is the brown dog tick, *Rhipicephalus sanguineus*. Diseases caused by these pathogens occur worldwide and are among the most commonly reported diseases in dogs (Taques et al. 2016; Vieira et al. 2011). Unlike *E. canis* and other tick-transmitted diseases, ingestion of infected ticks by dogs is the main route of transmission of *Hepatozoon* rather than through the feeding of the tick on the host. However, alternative routes have been suggested and reported for both pathogens (Aguiar et al. 2007; Ewing & Panciera 2003). Both ehrlichiosis and hepatozoonosis are manifested by a variety of clinical signs that may include, among others, fever, haemophilia, bone marrow failure and death in irreversible cases (Gondim et al. 1998; Mundim et al. 1994).

It is documented that the occurrence of these pathogens hinders the development of livestock sector, which contributes about 49% of agricultural output in South Africa (Terkawi et al. 2011). Furthermore, it is currently unknown whether South African domestic dogs carry zoonotic tick-borne pathogens (TBPs). Therefore, considering dogs as pets and the significance of livestock production in the South African economic landscape, in this study, we determined the occurrence and phylogenetic relationship of parasitic protozoan parasites and *Ehrlichia* infecting domestic animals in north-eastern KwaZulu-Natal (KZN).

## Material and methods

### Blood samples

Blood samples were collected from healthy cattle, sheep, goats and dogs in three local municipalities, namely, Mtubatuba, Big 5 Hlabisa and UmHlabuyalingana of the uMkhanyakude district (28°01′25″9 S, 32°17′30″30 E), KZN province, South Africa (Figure 1). A total of 208 blood samples were obtained from cattle (*n* = 109), sheep (*n* = 10), goats (*n* = 40) and dogs (*n* = 49). In these municipalities, rural communal farming is predominately practised and forms the main source of income in some households in the area. The owners of the sampled animals did not have any information about the age of the animals nor knowledge on the type of breed for goats and sheep. The cattle breed is Nguni. Sheep are not desired as domestic animals in this province because of cultural beliefs, and hence, only few were available during the sampling period.

**FIGURE 1:**
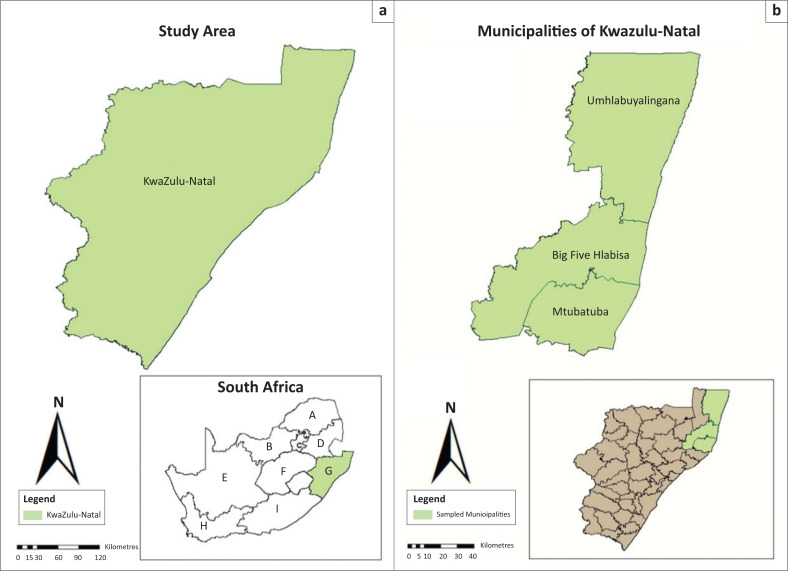
Map showing the sampled area. (a) KwaZulu-Natal province which is designated as G with the South African provincial map. (b) uMkhanyakude district with its three local municipalities that are in the northeastern part of KwaZulu-Natal province.

### Molecular detection of parasitic protozoa and Ehrlichia

Genomic deoxyribonucleic acid (DNA) was extracted using the salting out method adopted from Nasiri et al. (2005) with few modifications. Polymerase chain reaction was used to screen all the samples with genus or species-specific primers obtained from previous studies (Table 1). For each PCR assay, 2 *µ*L of the extracted genomic DNA was added into a 25-*µ*L reaction mixture containing 2.5 *µ*L of 10× standard *Taq* Reaction Buffer, 0.5 *µ*L of forward and reverse primer (10 *µ*M), 0.5 *µ*L of 10 mM Deoxynucleotide triphosphates (dNTPs), 0.125 *µ*L of *Taq* DNA polymerase and double distilled water (DDW) to a final volume to 25 *µ*L. The reactions were run on a proFlex thermocycler (Applied Biosystems, California, United States [US]) using the following thermocycling conditions: initial denaturation at 95 ºC for 30 seconds, followed by 35 cycles of denaturation at 95 ºC for 30 s. This was followed by annealing temperature (Table 1) for 1 minute, extension at 68 ºC for 1 min and final extension at 68 ºC for 5 min. Double distilled water was used as a negative control. Synthesised genomic DNA of *T. gondii* and canine *Babesia* referred to as g-block (Whitehead scientific-Integrated DNA Technologies, Johannesburg, South Africa) were used as positive controls for *Toxoplasma* and canine *Babesia*, respectively. The genomic DNA of *T. congolense* IL3000, *T. b. brucei* GuTat1.3 and *T. theileri* Japan Isolate was used as positive control for *Trypanosoma* species. The genomic DNA of *B. bigemina* South African strain, *B. bovis* SA strain obtained from North-West university, were used as positive control for bovine *Babesia*. The genomic DNA of *T. parva* provided by the North-West University was used as positive control for bovine *Theileria*. Following the amplification, 5 *µ*L amplicon was analysed by electrophoresis using 1% agarose gel stained with ethidium bromide and visualised under ultraviolet (UV) light. For nested PCR, 1 *µ*L of the primary PCR products was added into a second PCR mixture containing the same reagent composition as described above, except that the nested PCR primers were used instead of the external primers. Reaction mixtures were run as described above.

**TABLE 1:** Sequences of primers used for polymerase chain reaction amplification of protozoan parasites and *Ehrlichia canis.*

Species	Assay	Primer sequence (5′-3′)	Annealing (°C)	Product size	Target gene	Reference
*B. bovis*	PCR	CACGAGGAAGGAACTACCGATGTTGA CCAAGGAGCTTCAACGTACGAGGTCA	55	360 bp	*RAP-1*	Mtshali and Mtshali (2013)
*B. bovis*	nPCR	TCAACAAGGTACTCTATATGGCTACC CTACCGAGCAGAACCTTCTTCACCAT	55	298 bp		Mtshali and Mtshali (2013)
*B. bigemina*	PCR	CATCTAATTTCTCTCCATACCCCTCC CCTCGGCTTCAACTCTGATGCCAAAG	55	278 bp	*SpeI-AvaI*	Mtshali and Mtshali (2013)
*B. bigemina*	nPCR	CGCAAGCCCAGCACGCCCCGGTGC CCGACCTGGATAGGCTGTGTGATG	55	170 bp		Mtshali and Mtshali (2013)
*T. parva*	PCR	ATG ACA AAC ACA GAA GTC GCC CT ATT TCA TCC TTC TTC TTG ATT GCG T	53	1101 bp	18S SSU rRNA	Mans et al. (2011)
*Trypanosoma sp.*	PCR	GCG TTC AAA GAT TGG GCA AT CGC CCG AAA GTT CAC C	58	300 bp – 800 bp		Desquesnes and Davila (2002)
*Babesia/Theileria sp.*	PCR	CAC AGG GAG GTA GTG ACA AG AAG AAT TTC ACC TAT GAC AG	58	389 bp – 430 bp	18S rRNA	Shayan, Hooshmand and Rabhari (2008)
*B. ovis*	nPCR	GTC TGC GCG CGG CCT TTG CG CAC AGG GAG GTA GTG ACA AG	58	186 bp	18S rRNA	Shayan et al. (2008)
*B. motasi*	nPCR	CGC GAT TCC GTT ATT GGA G CAC AGG GAG GTA GTG ACA AG	58	205 bp	18S rRNA	Shayan et al. (2008)
*T. gondii*	PCR	TCT TTA AAG CGT TCG TGG TC GGA ACT GCA TCC GTT CAT GAG	63	194 bp	B1 gene	Burg et al. (1989)
*T. gondii*	nPCR	GGC GAC CAA TCT GCG AAT ACA CC TGC ATA GGT TGC AGT CAC TG	57	194 bp		Burg et al. (1989)
*B. rossi*	PCR	GTG AAC CTT ATC ACT TAA AGG AGG AGT TGC TTA CGC ACT CA	50	342 bp	18S rRNA	Duarte et al. (2008)
*B. vogeli*	PCR	GTG AAC CTT ATC ACT TAA AGG CAA CTC CTC CAC GCA ATC G	50	590 bp	18S rRNA	Duarte et al. (2008)
*E. canis*	PCR	TCG CTA TTA GAT GAG CCT ACG T GAG TCT GGA CCG TAT CTC AGT	60	154 bp	16S rRNA	Peleg et al. (2010)
*H. canis*	PCR	ATA CAT GAG CAA AAT CTC AAC CTT ATT CCA TGC TGC AG	57	625 bp	18S rRNA	Rubuni et al. (2005)

bp, base pair; rRNA, ribosomal ribonucleic acid; *B. bovis, Babesia bovis; B. bigemina, Babesia bigemina; B. ovis, Babesia ovis; B. motasi, Babesia motasi; T. ovis, Theileria ovis; T. gondii, Toxoplasma gondii; E. canis, Ehrlichia canis; H. canis Hepatozoon canis; B. vogeli, Babesia vogeli; B. rossi, Babesia rossi; PCR, polymerase chain reaction.*

### Sequence alignment and phylogenetic analysis

The PCR-generated fragments were sent to Inqaba Biotechnical Industries (Applied Biosystem, Johannesburg) for purification and direct sequencing in both directions. One to three individually amplified DNA fragments of each selected sample were sequenced. The obtained sequences were compared with similar sequences of the same pathogens from other regions of the world in GenBank. Deoxyribonucleic acid sequences were edited, aligned with Clustal W and visually checked in MEGA 7.0. The genetic distance (*p*-distance) of the sequences between taxa was also calculated using MEGA version 7.0. Phylogenetic analysis was performed using maximum likelihood method with 1000 bootstrap replicates to estimate the robustness of individual branches (Mtshali & Mtshali 2013).

## Statistical analysis

The proportions for 95% confidence intervals (95% CIs) were computed as CIs for proportions with binomial data using no continuity correction (Mtshali et al. 2013). This was calculated by hand using $P=Z\left(\frac{1-p}{n}\right)$.

### Ethical considerations

This study was approved by the Scientific committee of the Integrated Pest Management of North-West University as a no risk study (project number NWU-IPM-2017-003).

## Results

### Overall infection rate

A total of 208 domestic animals were sampled in this study and were screened for *T. ovis, Babesia* sp., *Trypanosoma* sp., *T. gondii, H. canis* and *E. canis.* The *T. ovis* had an overall prevalence of 6.0% (95% CI = ±6.58), *Trypanosoma* sp. 9.6% (95% CI = ±4.00), *T. gondii* 2.4% (95% CI = ±2.08), *B. bovis* 30.3% (±8.60), *B. bigemina* 22.02% (±7.46) and *E. canis* 40.8% (95% CI = ±13.72). One set of mixed infection was detected in this study, and *B. bigemina* and *B. bovis* were detected with an overall rate of 3.7% (±3.53). *Babesia ovis, B. rossi, B. vogelli, H. canis* and *T. parva* were not detected from their respective hosts.

### Infection rate based on hosts

It was observed that 18.35% (95% CI = ±7.45) cattle, 7.5% (95% CI = ±16.33) sheep, 0.0% (95% CI = ±0.0) goats and 13.61% dogs were positive for at least one pathogen (Table 2). In sheep, *T. ovis* was detected in 3/10 (30%) (95% CI = ± 28.42), and *Trypanosoma* sp., *T. gondii* and ovine *Babesia* sp. were not detected. On the contrary, in cattle, *T. gondii* was detected in 5/109 which is 4.58% (95% CI = ±3.92) and *Trypanosoma* sp. (18.35%). In dogs, no canine *Babesia* and *Hepatozoon* were detected, although *E. canis* was identified in 40.8% (95% CI = ±13.72).

**TABLE 2:** Overall infection rate of protozoan parasites and *Ehrlichia* from different hosts.

Host	Total samples	*Trypanosoma* sp.	*B. bovis*	*B. bigemina*	*B. ovis*	*B. motasi*	*T. ovis*	*T. gondii*	*E. canis*
Cattle	109	18.35% (±7.45)†	30.3% (±8.60)	22.02% (±7.46)	NS	NS	NS	4.58% (±3.92)	NS
Sheep	10	0.0% (±0.0)	NS	NS	0.0% (±0.0)	0.0% (±0.0)	30% (±28.42)	0.0% (±0.0)	NS
Goats	40	0.0% (±0.0)	NS	NS	0.0% (±0.0)	0.0% (±0.0)	0.0% (±0.0)	0.0% (±0.0)	NS
Dogs	49	0.0% (±0.0)	NS	NS	NS	NS	NS	0.0% (±0.0)	40.8% (±13.72)

NS, not screened.; *B. bovis, Babesia bovis; B. bigemina, Babesia bigemina*; *B. ovis, Babesia ovis*; *B. motasi, Babesia motasi*; *T. ovis, Theileria ovis; T. gondii, Toxoplasma gondii*; *E. canis, Ehrlichia canis.*

† , 95% confidence intervals.

### Infection rate based on the local municipalities

The highest occurrence of protozoan parasites and *E. canis* was recorded in Big 5 Hlabisa local municipality in which 30% (95% CI = ±28.42), 9.4% (95% CI = ±2.49), 12.64% (95% CI = ±7.81), 30% (95% CI = ±27.17), 30.3% (95% CI = ±10.92) and 6.19% (95% CI = ±18.11) of animals were infected with *T. ovis, T. gondii, Trypanosoma* sp., *E. canis, B. bovis* and *B. bigemina*, respectively. The uMhlabauyalingana local municipality had the highest infection rate followed by Big 5 Hlabisa, while the lowest prevalence was observed in Mtubatuba local municipality (Table 3).

**TABLE 3:** Infection rate of parasites from different municipalities.

Municipalities	Total samples	*Trypanosoma* sp. (%)	*Babesia bigemina* (%)	*Babesia bovis* (%)	*Theileria ovis*	*Toxoplasma gondii* (%)	*Ehrlichia canis* (%)
Big 5 hlabisa	129	12.64	6.19	30.3	30	5.38	30
uMhlabauyalingana	48	12.5	3.75	0.0	0.0	0.0	55.6
Mtubatuba	31	0.0	6.25	0.0	0.0	0.0	36.3

## Comparative analysis

The BLASTn analysis of the partial sequence of *RAP-1* genomic region (320 base pairs [bp]) of *B. bovis* obtained in this study (MN683993) matched with similar congeners from China (KT318580.1) and South Africa (KC894392) with high bootstrap support. The other sequence from this study (MN683992) showed 99.0% nucleotide identity with the above-mentioned strains. The *T. ovis* partial sequences (MK643268 and MK643269) of 18S ribosomal ribonucleic acid (rRNA) gene showed 98.4% nucleotide identity with other homologous sequences (MN493111.1 and MF182656.1) extracted from GenBank. One *B. bigemina SpeI-AvaI* gene sequence obtained from this study matched with LK391707.1 from the United Kingdom with identity of 89.0% and coverage of 88.0% (Figure 1-A1). The BLASTn analysis of the B1 gene sequence of *T. gondii* (194 bp) obtained in this study showed 100% nucleotide identity with homologous sequence of *T. gondii* from China (MK521884.1) and Mexico (KX270388.1). The BLASTn alignment with one of the sequences is indicated in supplementary Figure 2-A1. The BLASTn analysis of the 16S rRNA gene sequence of *E. canis* (124 bp) obtained in this study showed 89% nucleotide identity with homologous sequence of *E. canis* from Nigeria (JQ976640.1) (Figure 3-A1).

## Phylogenetic analysis

Retrieved sequences from the amplification of the *Rap-1* gene for detection of *B. bovis* were deposited to GenBank under the accession numbers MN683992 and MN683993. Subsequently, the maximum likelihood tree revealed three major clades with high bootstrap support values as well as divergence estimates (99% identical, *p* = 0.034) with an average distance of *p* = 0.053 (Figure 2 and Table 4). Sequences generated from this study (at 99% bootstrap support) formed a sister clade with the South Africa, China and Brazil sequences at 80% bootstrap. When constructing the phylogenetic tree, the two *T. ovis* 18S rRNA sequences from this study (MK643269 and MK643268) clustered into a single clade with *T. ovis* sequences from various countries including South Africa with 100% identity and average divergence between the species was *p* = 0.022 (Figure 3 and Table 5).

**FIGURE 2:**
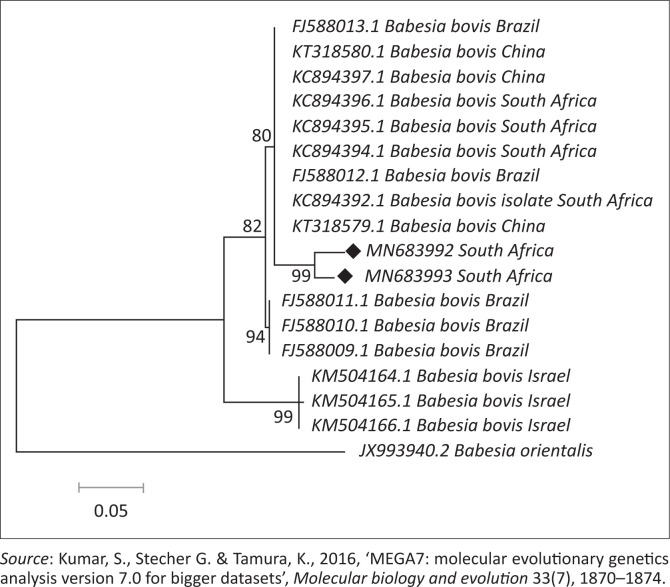
In phylogenetic tree analysis of *Babesia bovis* based on *RAP-1* gene, the tree was constructed with maximum likelihood method based on the Tamura-3-parameter model, with bootstrap values (expressed as percentages of 1000 replications) superimposed at branching points. The sequences produced in this study are shown with bullet points. The evolutionary distances were computed using the *p*-distance method. *Babesia orientalis* was used as an out-group.

**FIGURE 3:**
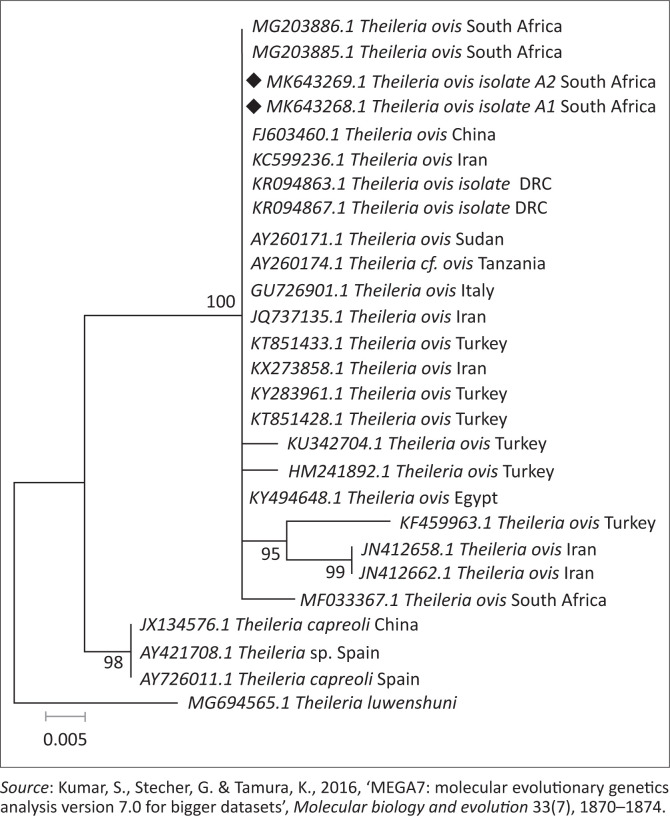
In phylogenetic tree analysis of *Theileria ovis* based on 18S ribosomal ribonucleic acid gene, the tree was constructed with maximum likelihood method based on Jukes–Cantor model, with bootstrap values (expressed as percentages of 1000 replications) superimposed at branching points. The sequences produced in this study are shown with bullet points. The evolutionary distances were computed using the *p*-distance method. *Theileria luwenshuni* was used as an out-group.

**TABLE 4:** Evolutionary divergence of *Babesia bovis* representing 320 base pair region and expressed as pairwise distance (*p*-distance) (bottom left) and standard error (top right).

Accession number and Origin	1	2	3	4	5	6	7	8	9	10
MN683992 South Africa	-	0.010	0.019	0.012	0.012	0.012	0.013	0.013	0.012	0.019
MN683993 South Africa	0.034	-	0.019	0.011	0.011	0.011	0.012	0.012	0.011	0.019
KM504165.1 Israel	0.147	0.135	-	0.016	0.016	0.016	0.016	0.016	0.016	0.003
FJ588012.1 Brazil	0.053	0.044	0.094	-	0.000	0.000	0.005	0.005	0.000	0.016
KC894395.1 South Africa	0.053	0.044	0.094	0.000	-	0.000	0.005	0.005	0.005	0.016
KC894395.1 South Africa	0.053	0.044	0.094	0.000	0.000	-	0.005	0.005	0.000	0.016
FJ588010.1 Brazil	0.063	0.053	0.091	0.009	0.009	0.009	-	0.000	0.005	0.015
FJ588009.1 Brazil	0.063	0.053	0.091	0.009	0.009	0.009	0.00	-	0.005	0.015
KT318580.1 China	0.053	0.044	0.094	0.000	0.000	0.000	0.009	0.009	-	0.016
KM504164.1 Israel	0.144	0.132	0.003	0.091	0.091	0.091	0.088	0.088	0.091	-

*Source:* Kumar, S., Stecher G. & Tamura, K., 2016, ‘MEGA7: molecular evolutionary genetics analysis version 7.0 for bigger datasets’, *Molecular biology and evolution* 33(7), 1870–1874.

**TABLE 5:** Evolutionary divergence of *Theileria ovis* representing 439 base pair region and expressed as pairwise distance (*p*-distance) (bottom left) and standard error (top right).

Accession number and Origin	1	2	3	4	5	6	7	8	9
MK643268.1 South Africa	-	0.000	0.007	0.007	0.004	0.004	0.013	0.010	0.000
MK643269.1 South Africa	0.000	-	0.008	0.009	0.006	0.006	0.013	0.010	0.000
HM241892.1 Turkey	0.012	0.019	-	0.007	0.004	0.005	0.014	0.012	0.006
MF033367.1 South Africa	0.015	0.023	0.019	-	0.004	0.005	0.014	0.013	0.007
MG203886.1 South Africa	0.004	0.011	0.006	0.012	-	0.003	0.013	0.010	0.000
MG203885.1 South Africa	0.004	0.011	0.009	0.015	0.004	-	0.013	0.010	0.000
KF459963.1 Turkey	0.044	0.045	0.053	0.058	0.044	0.044	-	0.012	0.013
JN412658.1 Iran	0.027	0.028	0.036	0.041	0.027	0.027	0.041	-	0.010
KY494648.1 Egypt	0.000	0.000	0.008	0.013	0.000	0.000	0.044	0.027	-

*Source:* Kumar, S., Stecher, G. & Tamura, K., 2016, ‘MEGA7: molecular evolutionary genetics analysis version 7.0 for bigger datasets’, *Molecular biology and evolution* 33(7), 1870–1874.

## Discussion

Protozoan and ehrlichial diseases are veterinary, medically and economically important contagious diseases affecting the domestic animals in sub-Saharan Africa, and hence, their prevalence and control is very important (Ademola & Onyiche 2013; Ringo et al. 2018). In this study, a low prevalence of protozoan parasites was observed and this could be attributed to the low sample size. It could also be because of the improvement in husbandry systems, better veterinary care and climate change.

There is no epidemiological data on livestock toxoplasmosis in this study area and, to the best of our knowledge, this is the first study that has detected *T. gondii* in cattle blood by PCR in South Africa. Recent serological studies have also reported a seroprevalence of 16.9%, 10.1% and 13.81% in cattle using latex agglutination test, Microscopic agglutination test (MAT) and enzyme-linked immunosorbent assay (ELISA), respectively, in Nigeria (Joshua & Akinwumi 2003; Okewole 2007; Onyiche & Ademola 2015). The low prevalence of *Toxoplasma* in cattle in the present study could be because cattle are genetically resistant to *T. gondii.* However, its presence could be attributed to the fact that they are raised outdoors as grazing animals to meet their nutritional needs, and as a result, they have more contact with rodents and soil contaminated with oocyst (Onyiche & Ademola 2015). Differences in the levels of infection with *T. gondii* between cattle in the different studies could possibly reflect variations in exposure rates to the parasite, which could be attributed to the contamination rate of the environment. No *T. gondii* DNAs were detected in the dog, goats or sheep samples, which contradicts reports from Ethiopia and Tunisia where *T. gondii* DNA was detected in 45.45% and 1.8% of goats and sheep samples, respectively (Gebremedhin et al. 2014; Gharbi et al. 2013). There are various risk factors such as age, sex, breed and climate conditions which may have contributed to the differences in prevalence in this study and other studies across the world.

Little attention has been given to ovine piroplasmosis compared to bovine piroplasmosis despite its widespread distribution through tropical and subtropical areas. According to Berggoetz et al. (2014), theileriosis in small ruminants can be caused by a number of well-known species such as *T. ovis, T. seperata* and *T. lestoquardi.* In this study, *T. ovis* was the only species detected (6.0%). However, previous studies in South Africa have reported *T. ovis* with a higher infection rate in small ruminants, whereby Ringo et al. (2018) reported an overall infection of 19.8% and Berggoetz et al. (2014) reported an overall infection of 10.9%. *Theileria ovis* known to be an agent of benign ovine and caprine theileriosis which has little economic importance (Mtshali et al. 2015) was identified in 30% of sheep and none of the goats in this study. It was suggested that there are two possible reasons for the higher prevalence of TBPs in sheep as compared to goats: firstly, detection of ticks can be hampered by too much hair, which covers the sheep, resulting in persistence and low awareness of TBPs in sheep. Secondly, differences in natural resistance against TBPs among sheep and goats could influence the prevalence of the parasites (Aydin, Aktas & Dumanli 2015, Gebrekidan et al. 2014; Rjeibi et al. 2014). Although the pathogen is less pathogenic, it cannot be completely neglected.

The phylogenetic tree of 18S rRNA gene sequences constructed in the present study revealed that *T. ovis* from this study was placed in the same clade with most of the *T. ovis* sequences in this tree.

The absence of ovine *Babesia* sp. in this study is similar to previous reports by Aktas, Altay and Dumanli (2007) and Ringo et al. (2018) who could not detect ovine *Babesia* sp. in Turkey and South Africa, respectively. Similar results were also reported in Tunisia where *B. motasi* was not detected (Rjeibi et al. 2016). *B. ovis* is considered to be one of the most important TBPs in small ruminants and its absence could also be an indication that the pathogen is not common in the study area. From documented literature, *B. ovis* has only been documented in northern African countries including Algeria and Tunisia (Aouadi et al. 2017; Rjeibi et al. 2014).

*Babesia bigemina* and *B. bovis* are the two economically significant species infecting cattle in southern Africa, and they have shown to be present in all provinces of South Africa (Bock et al. 2004; Mtshali & Mtshali 2013). Generally, the occurrence of both *B. bigemina* and *B. bovis* in the study area could be because of the presence and distribution of their tick vectors (Mtshali & Mtshali 2013). In South Africa, the only vector of *B. bovis* is known to be *Rhipicephalus (Boophilus) microplus*, in contrast *B. bigemina* is transmitted by three tick vectors: *R. (B) microplus, Rhipicephalus (Boophilus) decoloratus* and *Rhipicephalus evertsi evertsi* (De Vos et al. 1994; Mtshali & Mtshali 2013). Uncontrolled movement of cattle that usually occurs within the province could also be one of the factors for the prevalence of bovine *Babesia* sp. in all municipalities sampled.

From the phylogenetic tree constructed, it is clear that our isolates showed a close relationship with *B. bovis* strains from South Africa, Brazil and China. The conservation of nucleotide diversity observed among the *RAP-1* sequences has also been observed by Mtshali et al. (2013) and Ramos et al. (2012) in South Africa and Brazil, respectively. However, the isolates from this study formed a monophyletic grouping that is very distinct from that of other published *B. bovis* strains. According to Mtshali et al. (2013), this indicates the presence of micro-heterogeneities between the *RAP-1* sequences within *B. bovis* strains. It is also important to note that the South African sequence KC894394 was obtained from samples collected in Mpumalanga province (Mtshali & Mtshali 2013), hence the lack of 100% identity to those generated in the current study.

With regard to the analysis of *SpeI-AvaI* restriction fragment sequence of *B. bigemina* isolates, the highest nucleotide identity was 89.0%. It was recently discovered that the *SpeI-AvaI* nested PCR assay specific for the detection of *B. bigemina* DNA also amplified a homologous fragment derived from *Babesia ovata* (Sivakimar et al. 2012). Nevertheless, this may not be the case in the present study because the presence of *B. ovata* has not yet been reported in the country’s cattle (Mtshali et al. 2013; Yoshinari et al. 2013). To date, only five countries (Japan, Korea, China, Mongolia and Thailand) have reported the occurrence of *B. ovata* in cattle (Suh 1987; Sivakumar et al. 2012; Yoshinari et al. 2013).

*Theileria parva* is considered as the most significant theilerial species in sub-Saharan Africa and known to cause widespread morbidity and mortality in endemic areas. The absence of *T. parva* in the present study is comparable to results from a recent study in some parts of Nigeria where a 0,0% prevalence of the pathogen was reported (Okorafor & Nzeako 2014). However, results of this study were not comparable to a study by Yusufmia et al. (2010) who reported a *T. parva* prevalence of 6.7% in cattle from South Africa. However, observations of the current study were not really surprising as it is known that Corridor disease is mainly restricted to buffaloes in South Africa because of strict preventative measures of the government that aim to ensure that the parasite is not introduced to cattle (Yusufmia et al. 2010). The specimens from this study were also obtained from apparently healthy cattle. In addition, South Africa is considered free of *T. parva*, except in designated Corridor disease-infected areas such as the Kruger National Park and Hluhluwe-iMfolozi Park that contain various wildlife species.

Animal trypanosomiasis acts as a serious impediment to animal husbandry in all tsetse fly infested regions of sub-Saharan Africa (Nimpaye et al. 2011). In the present study, 18.3% of cattle showed the presence of *Trypanosoma* DNA in their blood. Uilenberg (1998), Van den Bossche et al. (2006) and Mamabolo et al. (2009) documented that tsetse fly vectors prefer cattle as their hosts as compared to other animals. The presence of *Trypanosoma* sp. in cattle is an indication that the cattle from uMkhanyakude district have encountered tsetse flies and its low prevalence may be attributed to the low sample size. These findings agree with a previous study by Mamabolo et al. (2009) who reported a *Trypanosoma* sp. prevalence of 18.4% in cattle in KZN. No *Trypanosoma* DNA was detected from goats, sheep and dogs, respectively. The absence of *Trypanosoma* in sheep and goats may be because of several factors such as the low tsetse feeding activity related to their small size and anti-feeding behaviour such as leg kicks and stamping, tail and ear flicks, head movement and skin rippling. According to Kniepert (1981), in communal grazing area, they attack cattle and leave most of the small ruminants uninfected. Canine African Trypanosomiasis (CAT) is seldomly reported (Gow, Simpson & Picozzi 2007; Keck et al. 2009). Recently, Lisulo et al. (2014) reported on the occurrence of CAT in Zambian dogs. To date, in South Africa, only two cases have been reported, the first case was documented by Gow et al. (2007) where a 6-year-old dog was infected with *T. congolense* and Matjila et al. (2008) reported a case of *T. congolense* in a dog sample from the northern parts of KZN. Generally, CAT caused by *T. congolense* is fatal, thus, it would not be easy to find dogs which are carriers.

Ehrlichiosis is considered one of the most economically significant infectious diseases affecting small ruminants in tropical and subtropical regions (Ringo et al. 2018). The prevalence (40.8%) of *E. canis* in the blood samples of dogs from the present study is higher than the prevalence of 19% for *E. canis* that was reported by Mtshali et al. (2017) from *R. sanguineus* ticks in South Africa. Other studies have reported seroprevalence of 42%, 34% and 53.8% for *E. canis* from dogs in South Africa, Zimbabwe and Namibia, respectively (Kelly, Eoghain & Raoult 2004; Manyarara et al. 2015; Pretorius & Kelly 1998). It is worth noting that the sampled dogs came from a low income area of KZN where there is a lack of access to effective tick control that could have contributed to the high prevalence.

Canine *Babesia* sp. and *Hepatozoon* sp. are causative agents of important tick-borne protozoal diseases of dogs. Despite the fact that dogs are increasing in numbers because of their different purposes in the country, less attention has been given to them by researchers as compared to other animals (Abdel-Rhman, Hegazy & Al-Gaabary 2015). Although none of the domestic dogs in our study were positive for *Babesia*, the infection is known to be common in domestic dogs in South Africa with a prevalence of approximately 10% and a low prevalence has also been reported in Lusaka, Zambia (Collet 2000; Nalubamba et al. 2011). Failure to detect *Babesia* sp. in domestic dogs sampled in this study could be because the sampled animals have little or no contact with wild dogs including the black-backed jackal (*Canis mesomelas*), a known natural host of *B. rossi* (Penzhorn et al. 2017), or vectors, or the prevalence was too low to detect with our sample size. At present, in South Africa, *B. vogelli* has only been detected from the Free State and Onderstepoort Veterinary Academic Hospital, which is an indication that *Babesia vogeli* infection is not as widely spread as *B. rossi* in South Africa (Matjila et al. 2004, 2008). The absence of *H. canis* in this study is in agreement with studies by Criado-Fornelio et al. (2003) and Matjila et al. (2008) who could not detect this pathogen in domestic dogs from Europe and South Africa, respectively. *H. canis* has been reported from South African domestic dogs but only in wildlife. As *H. canis* is transmitted by ingestion of ticks, its absence in domestic dogs may be attributed to the fact that domestic dogs do not feed on live prey which reduce the probability of ingesting infected ticks with their prey (Baneth, Samish & Shkap 2007). The parasite also seems to be present in high numbers in reticuloendothelial cells, so blood samples are unlikely to harbour-infected cells (Conceição-Silva et al. 1988).

In conclusion, the findings of this study have expanded our knowledge on the prevalence and phylogenetic similarity between protozoan parasites and *Ehrlichia* isolates of South African origin. To date, this is the first study to detect *T. gondii* infections in cattle using conventional PCR in South Africa.

## Figures and Tables

**FIGURE 1-A1:**
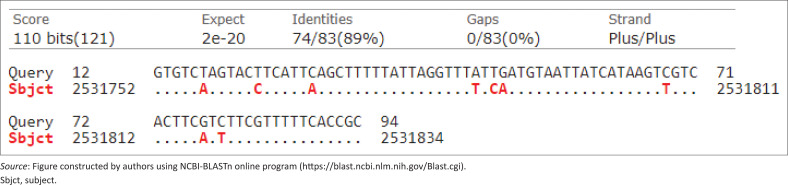
BLASTn result showing the alignment of *B. bigemina* isolate (United Kingdom – LK391707.1) and one sequence (*B. bigemina* KZN-Hlabisa isolate) obtained from the study. Nucleotide identity is represented by dots.

**FIGURE 2-A1:**
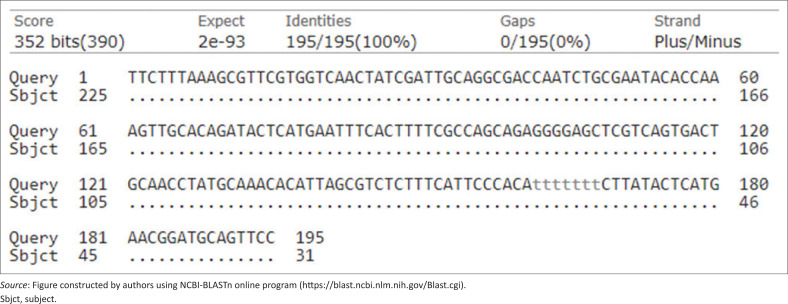
BLASTn result showing the alignment of *T. gondii* isolate (China –MK521884.1) and one sequence (*T. gondii* KZN-Hlabisa isolate) obtained from the study. Nucleotide identity is represented by dots.

**FIGURE 3-A1:**
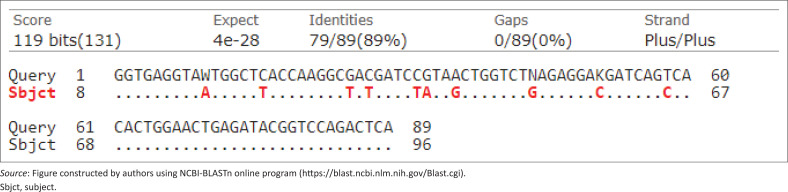
BLASTn result showing the alignment of *E. canis* isolate (Vietnam-MH686052.1) and one sequence (*E. canis* KZN-Hlabisa isolate) obtained from the study. Nucleotide identity is represented by dots.
